# Traditional Fermented Dairy Products as Reservoirs of *Bifidobacterium* With Probiotic Potential: From Microbial Diversity to Functional Characterization

**DOI:** 10.1111/1541-4337.70540

**Published:** 2026-06-22

**Authors:** Mst. Umme Habiba, Md. Morshedur Rahman, Mary Ann Augustin, Cristian Varela, Helen Morris, Hayriye Bozkurt

**Affiliations:** ^1^ School of Agriculture, Food and Wine Adelaide University, Waite Campus Urrbrae South Australia Australia; ^2^ Department of Dairy and Poultry Science, Faculty of Veterinary Medicine and Animal Science Gazipur Agricultural University Gazipur Bangladesh

**Keywords:** Bifidobacteria, functional foods, metagenomics, microbial diversity, probiotic potential, traditional dairy fermented products

## Abstract

Traditional fermented dairy products (TFDPs) are complex microbial ecosystems that may serve as reservoirs of many microorganisms, including those with probiotic potential such as *Bifidobacterium* species and lactobacilli. Although bifidobacteria are widely used as probiotic microorganisms in defined formulations, their occurrence, persistence, and functional relevance within TFDPs remain incompletely understood. This review critically synthesizes current evidence on the diversity, ecological roles, and traits associated with probiotic potential of *Bifidobacterium* spp. detected in TFDPs, including raw‐milk fermentations, artisanal dairy products, and selected controlled dairy systems. Species such as *Bifidobacterium animalis*, *Bifidobacterium longum*, *Bifidobacterium bifidum*, and *Bifidobacterium breve* have been reported across yogurt, kefir, airag (traditional Mongolian fermented dairy beverage from mare milk), and raw milk cheeses, often at low abundance or as transient microbial community members. Many isolates from fermented dairy products exhibit traits commonly associated with probiotic functionality, including acid/bile tolerance, adhesion capacity, exopolysaccharide production, and antimicrobial activity. However, most reports remain limited to presence/absence or in vitro assays, with limited in vivo or clinical validation. Advances in molecular and omics‐based approaches have improved detection, characterization, and safety evaluation; however, translation into validated applications remains constrained by challenges in isolation, viability, and strain‐level confirmation. Importantly, detection of bifidobacteria in TFDPs does not confer probiotic status, which requires strain‐level identification, demonstrated safety, adequate viable counts at consumption, and clinical evidence of health benefit. Collectively, TFDPs, as culturally embedded microbial reservoirs, may support the discovery of novel bifidobacterial strains for future development of functional foods or probiotic products following rigorous validation.

## 1 Introduction

Fermented foods represent complex and dynamic microbial ecosystems comprising bacteria, yeasts, and molds that originate from the natural microbiota of raw substrates; traditional utensils (e.g., wooden vats, fermentation jars, clay pots, bamboo baskets, grinding stones, ladles, and cutting boards); and the surrounding environment or intentionally added starter cultures (Franz et al. 2014; Lorenzo et al. 2018). These microbial consortia drive biochemical transformations that can enhance microbiological safety, stability, nutritional value, and sensory quality through acidification, production of antimicrobial metabolites such as organic acids and bacteriocins, and competitive inhibition of spoilage and pathogenic microorganisms (De Bellis and Rizzello 2024). However, the extent of safety improvement depends on raw material quality, hygienic practices, and control of fermentation conditions.

Many microorganisms present in fermented foods possess traits associated with probiotic potential. According to the International Scientific Association of Probiotics and Prebiotics (ISAPP), probiotics are defined as “live microorganisms that, when administered in adequate amounts, confer a health benefit on the host” (Hill et al. 2014). Importantly, probiotic designation requires strain‐level identification, demonstrated safety, evidence of clinical benefit, and viability at the time of consumption, criteria that most traditional fermented foods do not necessarily or consistently fulfill. Moreover, probiotic efficacy is highly strain‐specific and context‐dependent, and not all products containing live microorganisms confer measurable clinical benefits (Hill et al. 2014; Sanders et al. 2018). Although increasing consumer demand has driven rapid expansion of the probiotic market, this growth is accompanied by variability in product quality, regulatory definitions, and levels of scientific substantiation. These inconsistencies highlight the need for rigorous frameworks linking microbial identification to validated health outcomes.

Within this context, traditional fermented dairy products (TFDPs) should not be considered probiotic delivery systems per se but rather as ecological reservoirs for the discovery of bifidobacterial strains. In line with current regulatory frameworks, probiotic application requires strain‐level isolation, characterization, safety assessment, and clinical validation, followed by incorporation of validated strains into defined and controlled carrier systems (Hill et al. 2014; Saleena et al. 2024; Sanders et al. 2018). Accordingly, this review explicitly distinguishes between microbial presence within TFDPs and the subsequent development of validated probiotic strains to avoid misinterpretation of in situ microbial occurrence as evidence of probiotic functionality or regulatory compliance.

TFDPs represent a particularly important subset of fermented foods because of their microbial richness, cultural significance, and widespread consumption (de Oliveira et al. 2025; Hernández‐Velázquez et al. 2024). Produced using raw or minimally processed milk and artisanal practices such as back‐slopping (a portion of a previously fermented batch is added into fresh ingredients to initiate new fermentation), these systems support microbial transmission from the environment, dairy animals, equipment, and human handling. While such practices preserve ecological complexity and may facilitate microbial adaptation and niche specialization, they can also introduce potential safety risks if hygienic controls are inadequate (Anyogu et al. 2021). TFDPs are therefore best understood as complex microbial ecosystems rather than standardized functional foods (Franz et al. 2014; Kariyawasam et al. 2021), although they represent valuable sources for the discovery of candidate probiotic strains. The global probiotic food market exceeded USD 110 billion in 2025, reflecting rising consumer demand for foods containing beneficial live microorganisms and increasing interest in TFDPs as sources of novel probiotic strains (Caetano et al. 2024; Intel Market Research 2025).

Among microorganisms associated with fermented dairy systems, lactobacilli and bifidobacteria are the most extensively studied due to their technological adaptability and documented health‐related effects (Anjum et al. 2014; Latif et al. 2023; Sarita et al. 2025). Other genera containing strains with probiotic applications, including *Saccharomyces, Bacillus, Propionibacterium, and Streptococcus*, have also been isolated from fermented foods, though their occurrence and roles vary across substrates and processing conditions (Latif et al. 2023). Importantly, probiotic functionality remains strain‐dependent, with clinical studies demonstrating distinct physiological endpoints such as modulation of gut microbiota, enhancement of immune response, and inhibition of pathogenic organisms, including *Escherichia coli*, *Salmonella* spp., and *Listeria monocytogenes* (Raheem et al. 2021; Sanders et al. 2018; Vinayamohan et al. 2024).

Bifidobacteria, first isolated by Henri Tissier in 1899 from the feces of breastfed infants, are Gram‐positive, anaerobic bacteria widely recognized for their roles in gut health, immune modulation, and metabolic regulation (Bocchio et al. 2024; Laureys et al. 2016; Saturio et al. 2021). They are dominant members of the infant gut microbiota, where they metabolize human milk oligosaccharides (HMOs), supporting their ecological success during early life (Saturio et al. 2021; Turroni et al. 2017). In adults, they remain important contributors to gut homeostasis. Species such as *Bifidobacterium longum*, *Bifidobacterium bifidum*, and *Bifidobacterium breve* produce short‐chain fatty acids (SCFAs), enhance gut barrier function, and synthesize vitamins and other beneficial metabolites (Chen et al. 2021; Richmond et al. 2025; Turroni et al. 2014, 2022; Yao et al. 2021). Despite these documented functional attributes, their incorporation into food systems remains challenging due to sensitivity to oxygen, acidity, temperature fluctuations, and processing stresses, which can reduce viability during production and storage (Sibanda et al. 2024; Soares et al. 2023; Thomashoff et al. 2026; Zuo et al. 2020; Zhong et al. 2025).

The presence of bifidobacteria in dairy foods may originate from endogenous milk microbiota, environmental inputs, or intentional incorporation during processing (Jena and Choudhury 2025). However, they are often detected at low abundance in fermented dairy products, and their persistence within these systems remains uncertain (Firrman et al. 2025; Lee and O'Sullivan 2010).

In addition, competition with dominant lactic acid bacteria (LAB), which contribute to product safety and stability through acid production and antimicrobial activity, can further limit bifidobacterial survival (Choi et al. 2018; Shokryazdan et al. 2017; Zapaśnik et al. 2022).

Given the growing demand for probiotic‐enriched dairy products and the limited availability of functionally robust strains suitable for industrial application, TFDPs represent promising yet underexplored sources of bifidobacteria (Jena and Choudhury 2025; Prasanna et al. 2014). However, the transition from microbial detection to validated probiotic application remains a key challenge. To address this gap, this review adopts an ecological systems perspective, evaluating TFDPs as dynamic microbial environments in which bifidobacteria may function as transient members, niche specialists, or candidate strains for probiotic development.

Within this framework, three interconnected levels are considered: (i) ecological detection, referring to the presence and distribution of bifidobacteria within complex fermentation consortia; (ii) functional validation, encompassing viability, metabolic activity, and traits associated with probiotic functionality; and (iii) translational relevance, requiring strain‐level characterization, technological robustness, and clinical evidence of health benefit.

**FIGURE 1:**
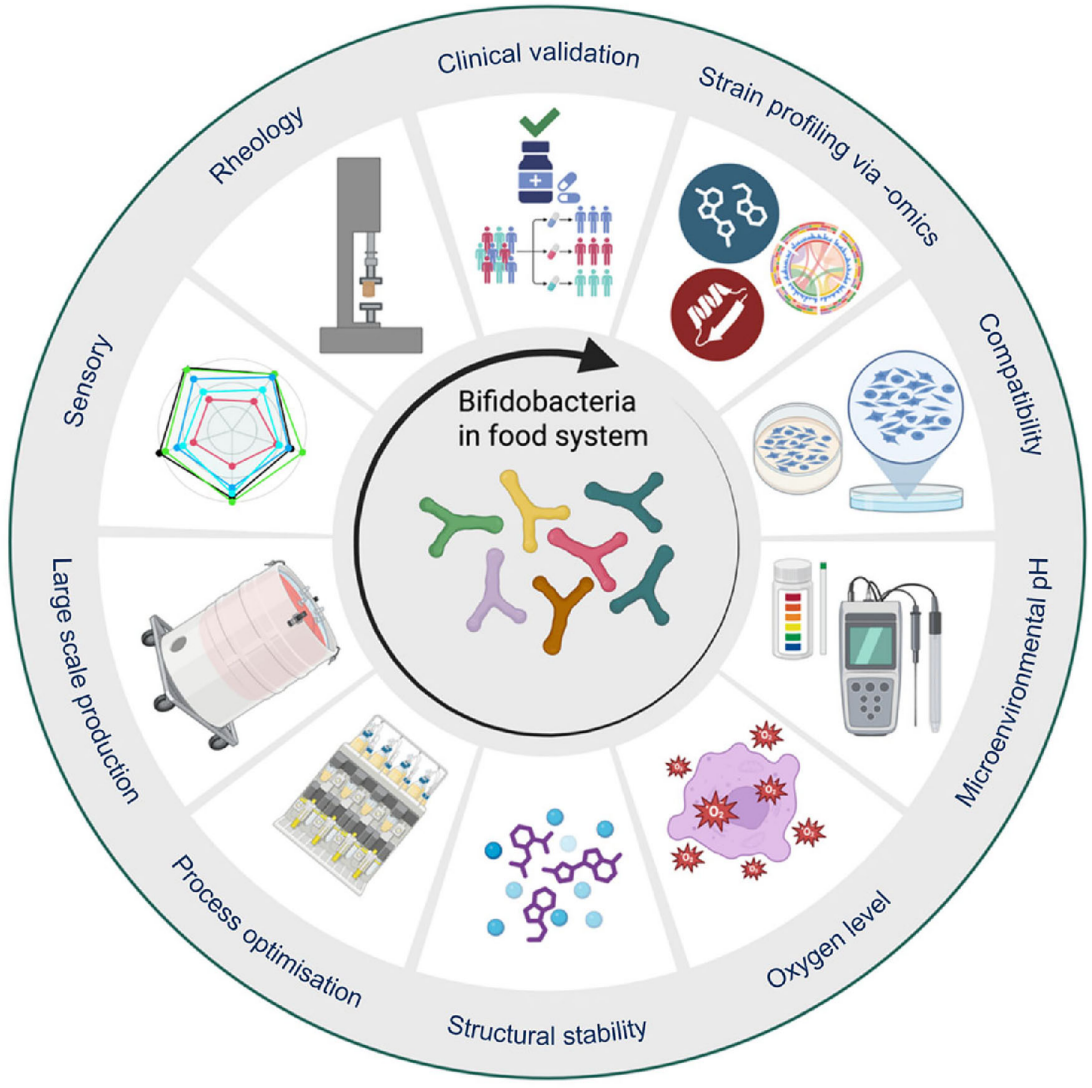
Conceptual overview of the major factors involved in the development of bifidobacteria in dairy foods. Molecular and omics‐based methods can substantially improve the precision with which bifidobacteria are detected, characterized, and assessed for safety in traditional dairy products. After careful strain selection, the chosen bifidobacteria are expected to be introduced into dairy matrices, where each factor shown in the figure should be evaluated to ensure optimal functionality, stability, and product safety.

This structured approach highlights the distinction between microbial presence and functional relevance and identifies key bottlenecks that limit the progression from detection to clinically validated probiotic applications (Figure 1).

## 2 Diversity and Microbial Ecology of Traditional Fermented Dairy Products (TFDPs): Implications for Bifidobacterial Discovery

Fermented dairy products (FDPs) are milk‐based foods that undergo microbial fermentation, during which LAB, yeasts, and occasionally molds drive biochemical transformations that influence digestibility, nutritional content, shelf life, and sensory characteristics. These microorganisms produce organic acids, antimicrobial compounds, and bioactive metabolites that shape product stability and quality and may contribute to functional properties associated with fermented foods, although such effects depend on microbial composition, viability, and host context (Gänzle 2015; Tamime and Robinson 2007).

TFDPs occupy a central role in regional diets worldwide and are characterized by artisanal practices, use of raw or minimally processed milk, and reliance on indigenous microbiota. These factors generate highly diverse microbial communities, including genera that contain strains with documented probiotic applications (Gebremichael et al. 2025; Sun et al. 2022). TFDPs therefore represent important dietary sources of diverse live microorganisms. Their metabolic activity may influence product characteristics and, in some cases, host physiology through metabolite production, immune modulation, or pathogen inhibition; however, such effects are product‐specific and strain‐dependent and not universally demonstrated (Dey et al. 2024; Kaindi et al. 2018).

Understanding the microbial composition and fermentation dynamics of TFDPs is essential for preserving traditional food systems and for identifying microorganisms with technological or probiotic potential (Xia et al. 2022). Factors such as fermentation practices, milk origin, and regional processing methods strongly influence community structure and stability (Table 1 ). Rather than representing uniform commercial products, TFDPs comprise heterogeneous ecological systems whose characteristics determine whether bifidobacteria can persist, interact with other microorganisms, and, critically, whether they can be reliably isolated as candidate strains for downstream functional and probiotic evaluation.

**TABLE 1:** Microbial profiles of traditional fermented dairy products (TFDPs) across regions.

Country/region		Fermentation type	Product	Milk type	Microbial highlights	References
Europe	Bulgaria, Greece, Turkey	Lactic acid	Yogurt	Cow milk	Fermented with *Lactobacillus delbrueckii* subsp. *bulgaricus* and *Streptococcus thermophilus*	Prajapati and Nair (2003); Tamime and Robinson (2007)
	Brittany region of France	Mixed yeast lactic acid	Gwell	Cow milk	*Lactococcus lactis* subsp. *cremoris, L. lactis* biovar *diacetylactis, Streptococcus* spp., *Geotrichum* *candidum* and *Kazachstania servazzii*	von Gastrow et al. (2020)
	Finland, Sweden	Mixed yeast lactic acid	Viili	Cow, goat milk; can be other types	*Lactococcus lactis*, *Leuconostoc* spp., and *G. candidum*	Bakry and Campelo (2018)
Middle East and Central Asia	Caucasus region, Russia, Turkey	Mixed acid alcoholic	Kefir	Cow, goat, sheep milk	*Lactococcus lactis* subsp. *lactis*, *S. thermophilus*, *Lactobacillus delbrueckii* subsp. *bulgaricus*, *Lactobacillus helveticus*, *Lacticaseibacillus casei* subsp. *pseudoplantarum*, *Lentilactobacillus kefiri*, and *Levilactobacillus brevis*	Bourrie et al. (2016); Guzel‐Seydim et al. (2011); Selhub et al. (2014); Wszolek et al. (2006)
	Kazakhstan	Lactic acid	Shubat	Camel milk	*Lactobacillus helveticus*, *L. delbrueckii* subsp. *bulgaricus*, and *S. thermophilus*	Afzaal et al. (2021); Shingisov and Alibekov (2017); Tang et al. (2020); Wszolek et al. (2006)
	Mongolia, Kazakhstan, Russia	Mixed yeast lactic acid	Koumiss	Mare milk	*Lactobacillus helveticus*, *Lentilactobacillus kefiranofaciens*, *Lactococcus lactis*, *Lactococcus raffinolactis*, and *Citrobacter freundii*, *Dekkera anomala*, *K*. *unispora*, *Meyerozyma caribbica*, *Pichia* spp., *K. marxianus*, and uncultured *Guehomyces* spp.	
South Asia	India, Bangladesh, Nepal, Pakistan	Back‐slopping lactic acid	Dahi	Cow/buffalo milk	*Limosilactobacillus fermentum*, *Lactococcus lactis*, *Lactbacillus acidophilus*, *Weissella confusu*, *L. bulgaricus*, *L. delbrueckii*, *L. lindneri*, *Leuconostoc mesenteroides* subsp. *cremoris*, *Leuconostoc falkenbergense*, *L. citreum*, *L. helveticus, Bifidobacterium* spp., *Enterococcus* spp.	Balamurugan et al. (2014); Habiba et al. (2021), Habiba et al. (2023, 2024a), Habiba et al. (2024b); Mudgal and Prajapati (2017); Rawat et al. (2022)
	India, Pakistan	Controlled lactic acid	Lassi	Cow/buffalo milk; combination of both cow and buffalo milk	*Lacticaseibacillus rhamnosus*, *Lactobacillus acidophilus* and *S. thermophilus*	
Africa	South Africa, Zimbabwe	Spontaneous/controlled lactic acid	Amasi	Mostly cow milk; goat milk minorly	*Lactococcus lactis, Lacticaseibacillus casei, Lacticaseibacillus paracasei, Lactiplantibacillus pentosus, Lactiplantibacillus plantarum, Leuconostoc pseudomesenteroides, Enterococcus faecalis, Acetobacter* spp*., Acetobacter johnsonii, Aeromonas sobria, Citrobacter freundii*, several uncultured bacteria	Adesulu‐Dahunsi et al. (2020); Ajibola et al. (2021); Maleke et al. (2021); Osvik et al. (2013); Sangoyomi et al. (2010); Walsh et al. (2017)
	South Africa, Namibia	Mixed yeast lactic acid	Fermented milk	Cow, buffalo, sheep, goat milk	*Lactiplantibacillus plantarum*, *L. delbrueckii* subsp. *lactis*, *L. helveticus*, *Lacticaseibacillus casei *subsp. *pseudoplantarum*, *Lacticaseibacillus casei*, *Lactococcus lactis*, *L. diacetylactis*, *Lactobacillus acidophilus*, *Leuconostoc mesenteroides* and *E. faecalis*	
	Northern part of Nigeria	Natural/spontaneous lactic acid	Wara	Cow milk	*Lactobacillus* spp., *Leuconostoc* spp., *Lactococcus* spp. and *Pediococcus* spp.	
	Ghana, Nigeria, West Africa	Natural/spontaneous yeast‐lactic	Nunu (fura de nunu)	Raw cow milk	*Limosilactobacillus fermentum*, *Lactiplantibacillus plantarum*, *Leuconostoc mesenteroides*, *L. helveticus*, *E. faecium*, and *E. italicus*, *Candida parapsilosis*, *C. rugosa*, *C. tropicalis*, *Galactomyces geotrichum*, *Pichia kudriavzevii*, and *Saccharomyces cerevisiae*	
Latin America	Brazil, Colombia, Venezuela	Lactic acid	Cuajada	Sheep, goat milk	*Lactobacillus* spp.*, Lactococcus* spp.*, Leuconostoc* spp.*, Pediococcus* spp.*, Streptococcus* spp.*, and Enterococcus* spp.	Dutra et al. (2016); Gänzle (2015); Medina et al. (2011)
	Brazil	Lactic acid	Coalhada	Any pasteurized or sterilized milk	*Lactococcus lactis* subsp. *lactis and L. lactis* subsp. *cremoris* being common	
China and East Asia	China	Mixed yeast lactic acid	Kurut	Mainly Yak milk; cow, goat, sheep milk also used	*Lactobacillus delbrueckii* subsp. *bulgaricus, S. thermophilus*, *Lacticaseibacillus casei*, *L. acidophilus*, *Lactiplantibacillus plantarum*, *L. helveticus*, *Lactococcus lactis*, several uncultured bacteria	Liu et al. (2012); Zhang et al. (2014)

**TABLE 2:** Key microbial communities involved in traditional fermented dairy products (TFDPs) and their association with bifidobacteria.

Major group	Fermentation type	Key species	Ecological role	Bifidobacteria association	References
Lactic Acid Bacteria	Natural/spontaneous; lactic acid	*Lactobacillus* spp. (*L. delbrueckii* subsp.*bulgaricus*, *L. acidophilus*, *Lactiplantibacillus plantarum*)	Key acidifiers; contribute to texture and aroma; dominate early fermentation stages	Produce metabolites (e.g., lactate) that support bifidobacterial growth; Bifidobacteria can use lactate to produce a variety of metabolites	Fernández et al. (2015); Sen et al. (2023)
		*Streptococcus thermophilus*			
		*Lactococcus lactis*			
		*Leuconostoc* spp.			
LAB & Yeast	Yeast lactic	*Saccharomyces cerevisiae*	Contributes to the overall fermentation process through their metabolic activity	Can produce prebiotics, which act as food for Bifidobacteria thus stimulating their growth	Tullio (2024); Yang et al. (2022)
		*Kluyveromyces marxianus*			
LAB & Mold	Mold lactic	*Geotrichum* *candidum*	Contribute to the breakdown of proteins and fats, and producing specific flavor and texture characteristics	Might produce compounds that could influence the growth or metabolism of bifidobacteria, or vice versa	Gobbetti et al. (2010); Voidarou et al. (2020); Yang et al. (2022)
		*Penicillium* spp. (*P. roqueforti*)			
Acetic Acid Bacteria	Acetic acid	*Acetobacter* spp.	Contribute to acidification and antimicrobial properties through oxidise alcohols; contribute to unique flavors	*Gluconacetobacter* spp. aids in the formation of exopolysaccharides and increases the biomass of kefir grains without negatively affecting the sensory properties of kefir or the microbial community (e.g. *Lactobacillus* spp., *Lactococcus* spp., yeasts, *L. acidophilus*, and *Bifidobacterium* spp.)	Afzaal et al. (2021); Özdemir et al. (2015); Yassunaka Hata et al. (2023)
		*Gluconobacter* spp.			
Propionic acid bacteria	Propionic acid	*Propionibacterium freudenreichii*	Contribute to production of propionic acid, B12 and carbon dioxide, which create the characteristic “eyes” in the cheese	Both can interact positively in the gut; inhibit undesirable bacteria and promote the growth of bifidobacteria; combining with bifidobacteria as starter culture helps to improve sensory properties of the fermented products	Khankhalaeva et al. (2017); Piwowarek et al. (2018); Thierry et al. (2011)

### 2.1 Microbial Drivers of Fermentation: A Framework for Assessing Bifidobacterial Niches

TFDPs can be ecologically classified according to the dominant fermentation processes, including lactic, yeast‐lactic, mold‐lactic, and propionic and acetic acid fermentations. While numerous fermented foods and beverages exist globally, this section focuses specifically on milk‐based fermentation systems, as these environments represent the primary matrices in which bifidobacteria have been investigated within traditional dairy ecosystems. Although originally based on sensory attributes or dominant taxa (Caetano et al. 2024; Voidarou et al. 2020), this classification also provides a functional framework for evaluating the ecological compatibility of *Bifidobacterium* spp. in diverse dairy systems.

Lactic acid fermentation, the most common type, is driven by LAB such as *L. delbrueckii* subsp. *bulgaricus*, *Streptococcus thermophilus*, and *Lactococcus lactis*. These organisms convert lactose to lactic acid, reducing pH and redox potential and creating mildly acidic and low‐oxygen conditions (Zapaśnik et al. 2022). While such environments may transiently support obligate anaerobes, culture‐based studies rarely recover viable bifidobacteria from products such as yogurt or dahi (a traditional fermented milk product similar to yogurt and widely consumed in South Asian countries), and detections are typically limited to low or inconsistent abundance, often detected only as DNA signals (Solís et al. 2010; Zapaśnik et al. 2022). This suggests limited ecological compatibility under standard fermentation conditions, likely due to slow growth rates and competitive exclusion (Prasanna et al. 2014).

Yeast‐lactic fermentations, such as kefir, koumiss (Central and East Asian TFDP from mare milk), and viili (Nordic fermented milk), feature metabolically diverse consortia of LAB and yeasts including *Kluyveromyces marxianus* and *Saccharomyces cerevisiae*. These systems generate lactic acid, ethanol, and CO_2_, supporting broader metabolic networks that facilitate micronutrient exchange and cross‐feeding (Afzaal et al. 2021). Yeasts may also reduce oxygen levels and release B vitamins and amino acids, which may support anaerobic microorganisms. These interactions may enhance ecological opportunity for bifidobacteria; however, stable detection remains limited, with metagenomic studies reporting low and inconsistent abundance (Marsh et al. 2013).

Mold‐lactic fermentations, typical of blue cheeses, introduce aerobic fungi like *Penicillium roqueforti*, which increase oxygen availability and drive extensive proteolysis and lipolysis during ripening (López‐Díaz et al. 2023). These conditions are generally unfavorable for bifidobacteria, which are highly sensitive to oxygen. As a result, viable bifidobacteria are unlikely to persist under typical conditions, and detections are often limited to DNA signatures that may not reflect metabolic activity or survival (Smid and Kleerebezem 2014).

Propionic and acetic acid fermentations, observed in Swiss cheeses and kefir‐derived products, present a more complex scenario. While *Propionibacterium freudenreichii* and acetic acid bacteria (AAB) such as *Gluconobacter* spp. may reduce oxygen tension and interact with LAB, they also produce inhibitory metabolites such as propionic acid and acetic acid, which can constrain bifidobacterial growth. Nonetheless, spatial heterogeneity within these systems, such as microaerophilic niches in cheese matrices, may permit limited survival (Liu et al. 2012; Rabah et al. 2018).

**FIGURE 2:**
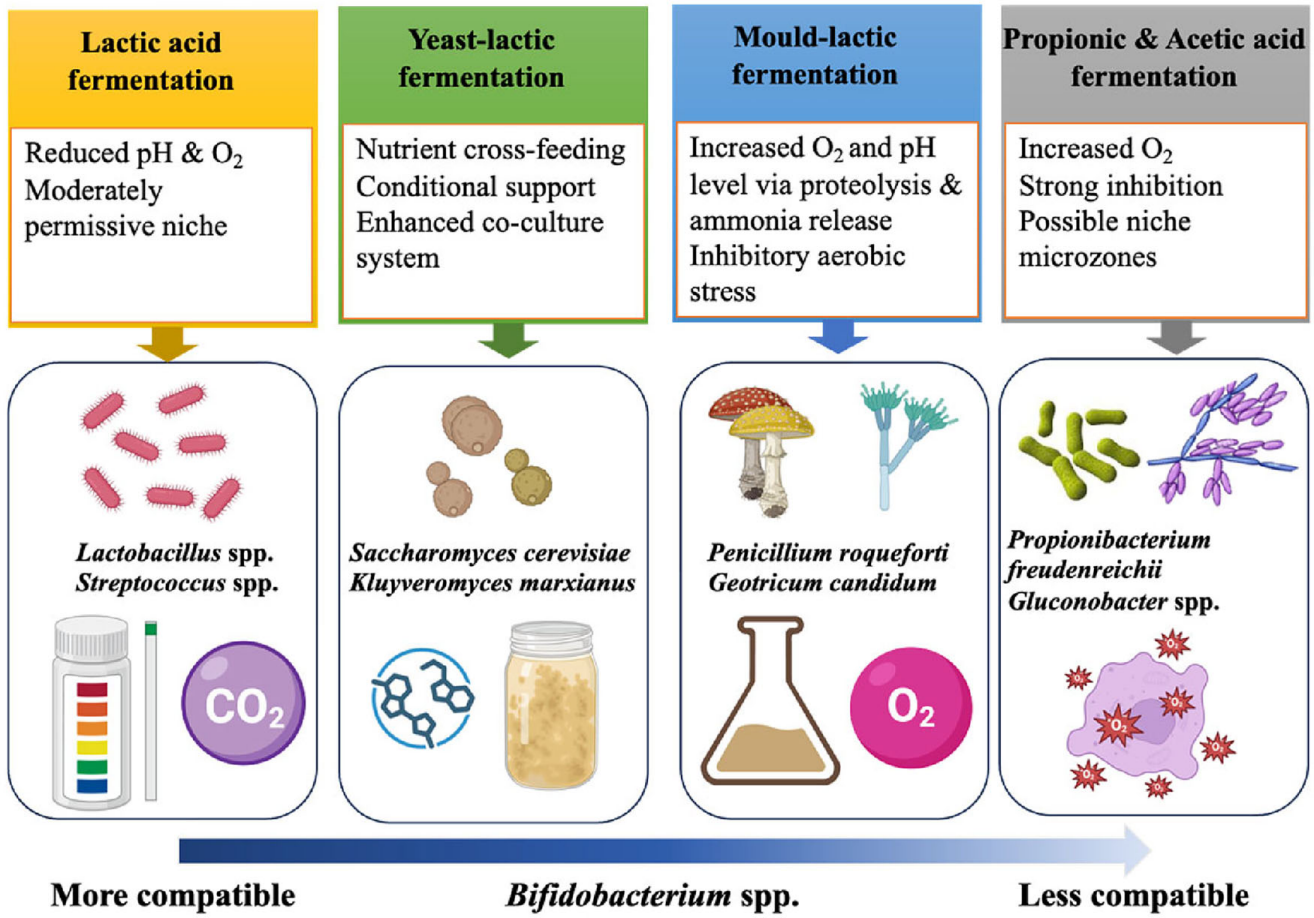
Ecological compatibility of traditional dairy fermentation systems with *Bifidobacterium* spp. Schematic representation of four major traditional dairy fermentation systems highlighting their ecological characteristics and compatibility with *Bifidobacterium* spp. The blue arrow represents the decreasing ecological compatibility for *Bifidobacterium* spp., integrating physicochemical and microbial dynamics.

Table 2 synthesises these ecological relationships by mapping key microbial groups in TFDPs with their known or proposed interactions with bifidobacteria. In addition, Figure 2 conceptualizes the ecological classification by illustrating how fermentation type influences bifidobacterial compatibility across key parameters, including pH, oxygen availability, microbial competition, and metabolite interactions. This framework highlights TFDPs as dynamic ecological systems in which environmental conditions, rather than simple microbial presence, determine the potential for bifidobacterial persistence and recovery.

### 2.2 Traditional Products as Microbial Reservoirs for Bifidobacteria: Contextualizing Regional and Process Variability

TFDPs exhibit a striking heterogeneity shaped by regional practices, raw milk microbiota, and artisanal processing techniques (Caetano et al. 2024; Tamang et al. 2016). While these differences are often highlighted to underscore cultural diversity, they also create distinct ecological contexts that may variably support or constrain bifidobacterial persistence. Understanding bifidobacteria within these systems therefore requires moving beyond geographic or typological descriptions toward a critical analysis of the microbial selection pressures that govern their occurrence.

Spontaneous fermentations, especially in rural or artisanal settings, typically rely on raw milk and environmental inoculation. These conditions generate complex microbial consortia and may facilitate the transient introduction or survival of bifidobacteria, including environmental or host‐associated strains. However, this microbial openness also introduces interpretative uncertainty. Reports of bifidobacteria in products such as artisanal dahi, labneh, or other raw milk‐derived fermentations are often difficult to contextualize, as post‐processing contamination from handling, water sources, or suboptimal hygiene conditions cannot be excluded (Kariyawasam et al. 2021; Mudgal and Prajapati 2017). Consequently, the detection of bifidobacteria alone cannot be interpreted as evidence of ecological integration within these systems.

Some fermented products, including shubat (fermented camel milk) and kurut (dried fermented milk shaped as balls), have shown preliminary evidence of compatibility as niches for bifidobacteria. However, these findings are frequently based on low‐resolution culture‐based methods or genus‐level molecular approaches, without sufficient strain‐level validation to confirm endogenous origin (Manaer et al. 2015; Afzaal et al. 2021; Shingisov and Alibekov 2017; Tang et al. 2020; Liu et al. 2012; Zhang et al. 2014). As a result, their ecological role, whether as commensal residents, transient populations, or contaminants, often remains unresolved.

A comparative synthesis of available studies (Table 1) underscores these methodological limitations. Many investigations lack standardized protocols for sample collection, enrichment, and strain typing, making cross‐study comparisons challenging. In addition, the geographic concentration of studies in South and Central Asian TFDPs leaves other traditional dairy systems underexplored. Critically, few studies incorporate metabolomic, transcriptomic, or viability‐based approaches to assess functional activity, leaving it unclear whether detected bifidobacteria are metabolically active or simply dormant or non‐viable.

To meaningfully position TFDPs as reservoirs of bifidobacteria, research must transition from sporadic detection to ecological validation. This requires integrated approaches combining strain‐resolved genomics to distinguish endogenous strains from contaminants, viability assays to confirm survival and metabolic activity, and longitudinal studies to assess persistence across fermentation batches and storage. Only through such integrated approaches can TFDPs be identified as genuine reservoirs of bifidobacterial strains with reproducible ecological persistence and demonstrable functional activity. These attributes must subsequently be confirmed through strain‐level isolation and validation prior to any consideration of translational application. This distinction is critical because stable or niche‐adapted populations are more likely to represent ecologically integrated strains with reproducible functional traits and greater potential for successful isolation and downstream probiotic development, whereas transient populations may reflect incidental presence without translational relevance.

Such integrative approaches, further elaborated in Section 4, provide a structured pathway for progressing from descriptive detection to evidence‐based identification of bifidobacterial strains with translational potential.

### 2.3 Microbial Interactions: Synergies, Competition, and Knowledge Gaps

Microbial interactions within TFDPs represent a critical determinant of whether bifidobacteria can persist, remain metabolically active, or be recoverable for downstream characterization. TFDPs are dynamic microbial ecosystems in which community structure and function are governed by complex inter‐microbial interactions rather than individual taxa alone (Gänzle 2015; Tamang et al. 2016). These interactions determine not only fermentation outcomes but also the capacity of specific microorganisms, including bifidobacteria, to persist or contribute functionally within the system. For bifidobacteria, ecological success depends on the balance between potentially supportive interactions and strong competitive or inhibitory pressures from dominant community members (Hill et al. 2014; Linares et al. 2017).

LAB dominate most TFDPs and play a central role in shaping the fermentation environment through rapid lactose metabolism, acidification, and reduction of redox potential (Zapaśnik et al. 2022). These changes may transiently create conditions that are more compatible with the oxygen‐sensitive physiology of bifidobacteria. In addition, LAB metabolism generates peptides, amino acids, SCFAs, B vitamins, or other metabolites that may act as co‐substrates or growth‐promoting factors (Gänzle 2015; O'Callaghan and van Sinderen 2016). However, these potentially beneficial effects are counterbalanced by strong competitive interactions. LAB typically exhibit faster growth rates, more efficient carbohydrate utilization, and the ability to produce antimicrobial compounds, such as bacteriocins or bacteriocin‐like inhibitory substances (BLIS), which can suppress slow‐growing anaerobes, including *Bifidobacterium* spp. Notably, bifidobacteria themselves have been shown to produce BLIS in dairy‐based media, underscoring the dynamic and bidirectional nature of these interactions (Balciunas et al. 2016; Martinez et al. 2015).

Several mechanistic processes have been proposed to explain how bifidobacteria may persist within TFDP ecosystems, although empirical support remains limited and context‐dependent. Cross‐feeding interactions are among the most widely suggested mechanisms, whereby proteolytic activity of LAB releases peptides, amino acids, and growth factors from casein hydrolysis that may support bifidobacterial metabolism (Gänzle 2015; Rivière et al. 2016). While such interactions are well documented in simplified co‐culture systems, their relevance in complex fermentation consortia remains uncertain, as competition for primary substrates often outweighs potential metabolic cooperation (Lee and O'Sullivan 2010; Prasanna et al. 2014).

Oxygen modulation represents another potential facilitating mechanism. Yeast and facultatively anaerobic LAB can reduce oxygen levels through respiratory activity, thereby creating microaerophilic niches (containing lower levels of dioxygen than atmosphere) that are more compatible with the strictly anaerobic nature of bifidobacteria (Afzaal et al. 2021). In yeast‐LAB co‐fermentations like kefir or koumiss, additional metabolic exchanges may occur, including the provision of vitamins (e.g., biotin and folate), amino acids, and redox‐active compounds that could alleviate physiological stress (Afzaal et al. 2021; Zhang et al. 2014). However, metagenomic studies consistently report low‐abundance and high variability of bifidobacteria in these systems, suggesting that such interactions are insufficient to support stable colonization (Marsh et al. 2013).

Matrix‐mediated protection has also been proposed as a mechanism influencing microbial persistence. Exopolysaccharide (EPS) production by LAB and other microorganisms can alter the physical structure of the fermentation matrix, potentially creating microenvironments that buffer pH fluctuations, limit oxygen diffusion, and enhance microbial stability (Gänzle 2015; Rivière et al. 2016). Such structural effects may transiently support the survival of oxygen‐sensitive microorganisms. However, direct experimental evidence linking matrix microstructure to sustained bifidobacterial viability in TFDPs remains limited.

Recent metabolomic studies have provided additional insight into microbial interactions by linking co‐culture activity to metabolite exchange within fermentation systems. Analytical approaches such as LC–MS and GC–MS have demonstrated that mixed fermentations can result in increased concentrations of SCFAs, free amino acids, and bioactive peptides compared to single‐strain systems (Rivière et al. 2016; Afzaal et al. 2021). These metabolites may serve as secondary substrates or signaling molecules influencing bifidobacterial metabolic activity (O'Callaghan and van Sinderen 2016). However, most metabolomic findings remain correlative, and there is still limited direct evidence linking specific metabolite fluxes to bifidobacterial growth dynamics or functional expression within complex TFDP matrices.

Despite these mechanistic insights, it is important to recognize that most supporting evidence originates from simplified in vitro or model systems that do not fully replicate the ecological complexity of TFDPs. In real TFDP ecosystems, potentially cooperative interactions are frequently outweighed by competitive pressures, including rapid acidification, substrate depletion, and antimicrobial activity of dominant microorganisms (Gänzle 2015; Zapaśnik et al. 2022). Consequently, bifidobacteria are often detected at low abundance and more likely to represent transient or residual populations rather than stable, ecologically integrated members of the microbial community.

Importantly, the presence of microbial interactions or co‐occurring taxa does not imply stable ecological integration or functional contribution of bifidobacteria within TFDPs. Distinguishing between transient occurrence and sustained ecological adaptation is therefore essential, particularly in the context of identifying strains with potential for isolation and downstream application.

Overall, while microbial interactions in TFDPs provide a theoretical basis for bifidobacterial survival, empirical evidence supporting consistent persistence or functional integration remains limited. Their ecological success is highly context‐dependent and rarely sustained under typical TFDP conditions, reinforcing the view that bifidobacteria are more often transient members of these systems rather than stable components.

## 3 Presence and Relevance of Bifidobacteria in Traditional Fermented Dairy Products (TFDPs)

Bifidobacteria have been intermittently reported in a range of TFDPs, including yogurt, kefir, airag, and raw‐milk cheeses, particularly those produced through spontaneous or artisanal fermentation (Table 3). Although earlier microbiological surveys frequently overlooked these taxa, recent metagenomic and culture‐dependent studies (summarized in Table 3) have improved the resolution of detection methods and, in some cases, enabled recovery of viable isolates from naturally fermented dairy systems. However, their occurrence is typically sporadic, low in abundance, and inconsistent across studies. While certain fermentation or storage conditions may permit survival or transient persistence, such occurrences rarely indicate stable ecological integration within the fermentation community. Moreover, the presence of bifidobacteria does not exclude contamination or the identification of non‐viable cells, and therefore requires careful ecological interpretation.

**TABLE 3:** List of traditional fermented dairy products (TFDPs) containing Bifidobacteria: Their sensory attributes, microbial consortia, and functional implications.

Country/region	Animal source	Name of TFDP	Fermentation type	Key microorganisms	Detection method	Sensory property and nature	Role of bifidobacteria	Functional insights	Limitations	References
Europe, Middle East	Cow Milk	Fermented cheese	Natural/spontaneous/back‐slopping lactic acid	*Lactococcus lactis*,* Bifidobacterium bifidum*,* Lactobacillus acidophilus*, *Lacticaseibacillus paracasei*	Preidentified strains were used; identification method not available	Soft or hard, solid; side dish, salad, used in many cooked/baked dishes	Act as primary player with probiotic potential	in vivo gut mucosal immunomodulation	Other functional and technological parameters not explored	Cuamatzin‐García et al. (2022); Medici et al. (2004)
Italy		Parmesan cheese	Natural/Spontaneous/back‐slopping lactic acid	*Bifidobacterium mongoliense*, *B. adolescentis*, *B. bifidum*, *B. breve*, *B. crudilactis*, *B. longum subsp. longum*, *B. pseudolongum* subsp*. globosum, B. animalis* subsp. *lactis*	16S rRNA/ITS microbial profiling	Hard, granular texture with a rich, nutty, and umami flavor, savory aroma and salty taste	Act as minor player, however, participate in the development of the organoleptic features of cheese	Data were not available	Metagenomics study; functional and technological parameters not explored; No* in vivo* validation	Milani et al. (2019)
France		Tomme d'Orchies cheese	Natural/spontaneous lactic acid	*Lactococcus* spp., *Streptococcus* spp.*, Lactobacillales*, *Corynebacterium* and *Brevibacterium, Bifidobacterium*, and *Micrococcales*	Metagenetic analysis using Illumina technology	Semi‐soft creamy texture, strong pungent aroma				Ceugniez et al. (2017)
Azores, Portugal		Pico cheese	Natural/spontaneous/back‐slopping lactic acid	Four phyla (Firmicutes, Proteobacteria, Actinobacteria and Bacteroidetes) and 54 genera were identified including *Bifidobacterium* genus	Pyrosequencing analysis	Soft, creamy, yellowish, mildly compact, unctuous paste with mild, tangy flavor	Secondary player	Data were not available	Metagenomics study; functional and technological parameters not explored; no in vivo validation	Riquelme et al. (2015)
Mexico		Water kefir	Symbiotic	*B. aquikefiri*, *Liquorilactobacillus harbinensis*, *Lentilactobacillus hilgardii*, *Liquorilactobacillus nagelii*, *Lacticaseibacillus paracasei*, *Lactobacillus* spp. similar to *Liquorilactobacillus* *hordei/mali* and two yeast species, namely *S. cerevisiae* and *Dekkera* (*Brettanomyces*) *bruxellensis*	Shotgun metagenomics	Slightly fizzy, sour, and acidic taste, with a fruity sweetness	Minor player	Data were not available	Shotgun metagenomics sequencing	Verce et al. (2019)
United Kingdom, Canada and United States			Symbiotic	*Zymomonas, Lactobacillus, Leuconostoc*, *Acetobacter, Gluconacetobacter* and Bifidobacteriaceae	Pyrosequencing analysis		Minor player	Data were not available	Metagenomics study; functional and technological parameters not explored; no in vivo validation	Marsh et al. (2013)
Germany			Natural/spontaneous	*Acetobacter*, *Gluconobacter*, *Gluconacetobacter*, *Bifidobacterium* (*B. psychraerophilum, B. crudilactis, B. subtile*), *Lactobacillus*, *Leuconostoc*, *Clostridium*	16S rRNA gene amplicon sequencing and amplified ribosomal DNA restriction analysis (ARDRA)		Dominant player	Data were not available	Metagenomics study; Functional and technological parameters not explored; no in vitro and in vivo validation	Gulitz et al. (2013)
Ireland			Natural/spontaneous	*Bifidobacterium fermentum* sp. nov. and *B. aquikefiricola* sp. nov.	16S rRNA gene analysis; Shotgun metagenomics		Minor player	Data were not available	Metagenomics study; Functional and technological parameters not explored; no in vitro and in vivo validation	Breselge et al. (2024)
Bhola, Bangladesh	Buffalo milk	Traditional buffalo milk curd	Natural/spontaneous	*Bifidobacterium* spp., *Leuconostoc citreum*, *Leuconostoc falkenbergense*	Culture dependent and PCR amplification	Mild‐acidic, sour with thick consistency, savory	Secondary player	Data were not available	No species‐specific identification; No in vitro and in vivo validation	Habiba et al. (2021, 2024a, 2024b)
Mongolia	Mare; camel milk	Airag	Natural/spontaneous	*Lactobacillus helveticus*, *L. kefiranofaciens*, *B. mongoliense*, *Kluyveromyces marxianus*	Culture dependent and independent approach (16S rRNA and ClpC ATPase (clpC) gene and fructose‐6‐phosphate phosphoketolase (F6PPK)	Acidic, sour, mild alcoholic drink	Act as primary player in the fermentation	Data were not available	No in vitro and in vivo validation	Watanabe et al. (2009); Yu et al. (2015)
Kalmykiya, Buryats and Tuva region of Russia	Mare; cow milk	Fermented milk	Back‐slopping method	Seven *Lactobacillus* species (*Lacticaseibacillus* *paracasei*, *Lacticaseibacillus* *fermentum*, *Lac* *tilactobacillus* sakei, *Lactobacillus acidophilus*, *Lactiplantibacillus* *plantarum*, *L. helveticus*, *L. delbrueckii* ssp. *bulgaricus*) and *Bifidobacterium* spp.	Quantitative PCR	Creamy texture, tangy flavor, and rich aroma, reflecting their natural fermentation and artisanal character	Act as secondary player in the fermentation	Data were not available	Functional and technological parameters not explored; no in vivo validation	Yu et al. (2015)
Northern Senegal		Fermented milk (lait caillé)	Natural/spontaneous	Predominately *Streptococcus*, *Lactobacillus*, *Lactococcus*, *Enterococcus*, *Bifidobacterium*, and *Bacillus* spp.	Bar‐coded 16S rRNA amplicon sequencing				Metagenomics study: Functional and technological parameters not explored; no in vitro and in vivo validation	Parker et al. (2018)
Croatia	Sheep milk	Croatian Cheese (Croatian raw ewe's milk cheeses)	Natural/spontaneous	*Lactobacillus*, *Streptococcus*, *Leuconostoc*, *Bifidobacterium*, *Brevibacterium*, *Corynebacterium*, *Clostridium*, *Staphylococcus*, *E. coli*, *Hafnia*, *Thermoanaerobacterium*, *Pseudomonas*, *Janthinobacterium*, *Petrotoga*, *Kosmotoga*, *Megasphaera*, *Macrococcus*, *Mannheimia*, *Aerococcus*, *Vagococcus*, *Weissella* and *Pediococcus*	Next generation 16S rRNA gene amplicon sequencing	Soft, rich, robust flavors and creamy texture	Secondary player	Data were not available	Metagenomics study; Functional and technological parameters not explored; no in vivo validation	Fuka et al. (2013)
Poland		Oscypek	Natural/ Spontaneous	*Lactococcus lactis subsp. lactis*, *Lacticaseibacillus casei, Leuconostoc citreum*, *Lactiplantibacillus plantarum, Leuconostoc lactis*, *Leuconostoc mesenteroides*, *S. thermophilus*, *Enterococcus faecalis*, *E*. *durans*, *Bifidobacterium* sp*., E*. *italicus*, *L. pseudomesenteroides*, *Lactococcus lactis subsp. cremoris*, *Bacillus simplex*, *L. parabuchneri, L. brevis*, *Enterobacter kobei*	Culture dependent and independent approach (PCR amplification and denaturing gradient gel electrophoresis (DGGE analysis); next generation sequencing (16S rRNA‐based pyrosequencing analysis)	Semi‐hard cheese with a distinctive flavor and texture	Secondary player	Data were not available	Functional and technological parameters not explored; no in vitro and in vivo validation	Alegría et al. (2012)
Slovakia		Ovine cheese	Natural/ Spontaneous	*Bifidobacterium crudilactis* and *B. animalis* subsp. *lactis*	Matrix‐assisted laser desorption/ionization time‐of‐flight analysis (MALDI‐TOF) and sequencing of phylogenetic three markers (16S rRNA, heat‐shock protein 60 kDa (hsp60), and elongation factor EF‐G (fusA)	Rich, creamy texture and a distinctively tangy, slightly nutty flavor that reflects its unique sensory properties and artisanal nature	Secondary player	Data were not available	Functional and technological parameters not explored; no in vitro and in vivo validation	Bunesova et al. (2014)
France		Yogurt and Priobiotic drinks	Symbiotic	*Lacticaseibacillus casei, Lactococcus lactis, B. animalis, Lactobacillus delbrueckii*, and *S. thermophilus*	MALDI‐TOF mass spectrometry	Acidic, thick‐gel viscous, curd‐like product and savory	Secondary player	Data were not available	Functional and technological parameters not explored; no in vitro and in vivo validation	Angelakis et al. (2011); Tamime and Robinson (2007)

Beyond their microbiological interest, TFDPs carry deep cultural and nutritional significance across Asia, Africa, and Europe, serving both as dietary staples and income sources in rural communities (Agyei et al. 2020; Mathara et al. 2004; Owusu‐Kwarteng et al. 2017). From a scientific perspective, understanding the occurrence and behavior of bifidobacteria within these traditional systems is relevant for strain discovery and probiotic bioprospecting. However, this does not imply that TFDPs themselves function as probiotic products. Rather, detection within these matrices should be interpreted as an indicator of potential strain sources, requiring subsequent isolation, characterization, and validation before any functional or health‐related claims can be made.

### 3.1 Ecological and Process‐Related Factors Influencing Bifidobacterial Persistence

While detection of bifidobacteria in TFDPs confirms of their presence, it does not necessarily indicate ecological adaptation or functional relevance. Distinguishing whether these microorganisms are transient, contaminant, or actively integrated members of the fermentation ecosystem requires careful evaluation of the environmental and technological conditions that govern their survival and persistence. This distinction is critical for assessing whether TFDPs can serve as potential reservoirs of bifidobacteria relevant to strain discovery and probiotic development.

The distinction between transient and stable populations is not merely ecological but has direct implications for strain discovery and selection. Stable or niche‐adapted populations are more likely to exhibit functional integration, technological robustness, and reproducibility, making them stronger candidates for downstream isolation and probiotic development, whereas transient populations may reflect environmental contamination or short‐term survival without functional or translational relevance (Sanders et al. 2018).

Bifidobacterial survival within dairy environments depends on a complex interplay between raw‑milk ecology, fermentation dynamics, and post‑processing conditions. Key determinants include the initial microbial load, hygienic practices during milking, fermentation parameters (pH, temperature, and oxygen), and the nutritional composition of the milk matrix.

The milk substrate plays a defining ecological role. Protein, fat, and carbohydrate composition influence microbial adhesion, metabolism, and acid tolerance. For example, buffalo milk, with its higher protein and lipid content, has been reported to support improved bifidobacterial survival compared with cow milk, possibly due to enhanced buffering capacity and substrate availability (Habiba et al. 2025; Linares et al. 2017). Nonetheless, most isolates characterized to date originate from cow‑milk‑based TFDPs, with few investigations targeting buffalo milk and even fewer focusing on camel milk (Table 3).

Environmental factors such as oxygen tension and acidity critically constrain *Bifidobacterium* viability. Bifidobacteria generally exhibit optimal growth in low‑oxygen, moderately acidic conditions (pH 4.5–6.5) and temperatures of 30°C–40°C (Roy 2001). Excessive acidification or prolonged aeration can reduce viable counts. Adaptation strategies include co‑culturing with oxygen‑scavenging LAB, microencapsulation, and modifying fermentation times to optimize micro‑niche conditions (Sionek et al. 2024; Wendel 2022). However, inconsistencies persist between laboratory and real‑world fermentation outcomes, with commercial products often showing variable retention of viable bifidobacteria at the end of shelf life (Shah 2000; Hang et al. 2026).

**FIGURE 3:**
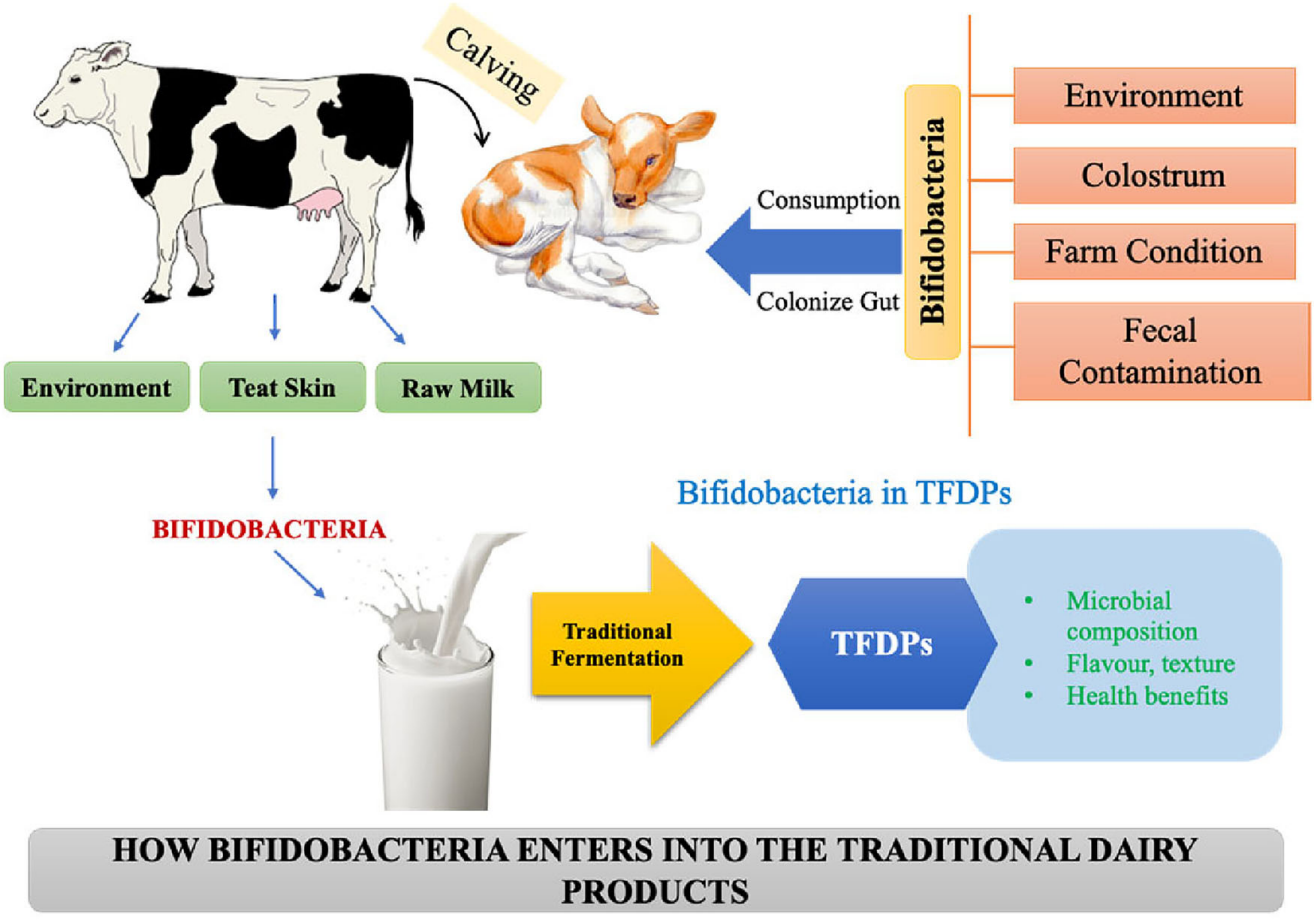
Proposed ecological routes of bifidobacterial transmission in traditional dairy systems, illustrating environmental, maternal, and process‑driven pathways contributing to microbial continuity from the farm ecosystem to the fermented product.

In traditional dairy ecosystems, bifidobacteria may enter via multiple routes, including maternal transmission (colostrum), environmental contact (teat surface, equipment, housing), or faecal shedding. While such transmission pathways of *Bifidobacterium* are well‐documented in humans (Milani et al. 2017; Mitsuoka 1984), evidence in ruminants remains limited and largely inferred from milk microbiome studies (Oikonomou et al. 2012). Once introduced, these microbes can persist through practices such as back‑slopping or continuous fermentation, where microbial consortia are recycled between batches. However, such practices also support microbial variability and contamination risk. Figure 3 illustrates these transmission pathways, depicting the cyclical link between animal microbiota, environmental exposure, and milk fermentation. This continuity mirrors broader host‑microbiome dynamics: the mammary gland, once assumed sterile, is now recognized as a microbial niche (Martín et al. 2007; Oikonomou et al. 2012). As these host‐associated microbial populations can be transferred into raw milk and subsequently carried into fermentation processes, traditional dairy fermentations may represent secondary reservoirs of host‑associated commensals, including bifidobacteria, bridging the animal gut‐milk‐fermentation continuum. Recognizing this ecological linkage helps explain how host‐associated microorganisms can enter dairy fermentation systems and provides a biological basis for investigating bifidobacteria within TFDPs.

Over time, some dairy‑associated *Bifidobacterium* strains have undergone ecological domestication, adapting to the milk environment and later being incorporated intentionally as components of commercial probiotic formulations. Isolates such as *B. longum* and *B. animalis* demonstrate survival through cheese ripening and transient detection in the human gut following consumption (Milani et al. 2019). This progression from spontaneous to controlled fermentation underscores the translational potential of TFDP‑derived bifidobacteria.

### 3.2 Functional and Technological Properties of Bifidobacteria in Traditional Fermented Dairy Products (TFDPs)

Bifidobacteria may influence both health‑related, nutritional, and technological aspects of TFDPs, and some strains exhibit traits commonly associated with probiotic functionality (He et al. 2022; Jena and Choudhury 2025). However, the presence of such traits in vitro does not establish probiotic status or clinical efficacy, which requires strain‐level identification, adequate viable dose at consumption, and evidence of health benefit in humans. Within TFDP contexts, bifidobacteria are therefore more appropriately discussed in terms of functional attributes and technological performance rather than confirmed probiotic effects.

Many isolates exhibit varying degrees of acid and bile tolerance, characteristics often used as preliminary indicators of potential survival through the gastrointestinal tract (Sánchez et al. 2013). Certain strains adhere to intestinal epithelial cells in vitro, suggesting possible host interaction (García‐Cayuela et al. 2014; Jungersen et al. 2014). In addition, some strains produce organic acids and antimicrobial compounds and have been reported to inhibit pathogens such as *E. coli*, *Staphylococcus aureus*, and *Salmonella typhimurium* under laboratory conditions (Collado et al. 2007), although their effectiveness in complex food matrices is strain‐specific and context‐dependent.

Beyond potential host interactions, bifidobacteria may contribute directly to product quality. EPS production can enhance viscosity, mouthfeel, and water‑holding capacity, thereby improving sensory properties. EPS may also confer stress protection, supporting bacterial survival during fermentation and storage (Hidalgo‐Cantabrana et al. 2014). Their enzymatic activities promote protein and carbohydrate hydrolysis, generating bioactive peptides and volatile compounds that influence flavor and produce metabolites associated with potential health benefits, such as SCFAs and, in some strains, γ‐aminobutyric acid (GABA) (Pokusaeva et al. 2011; Rivière et al. 2015).

Taken together, available evidence suggests that bifidobacteria detected in TFDPs may, under certain conditions, contribute to product quality rather than being solely incidental members of the microbiota. Nevertheless, the extent of their activity, persistence, and functional significance requires confirmation through strain‐resolved, viability‐based, and matrix‐specific investigations under realistic fermentation conditions.

### 3.3 Evidence From Isolates: Probiotic Screening of **T**FDP‐Derived Bifidobacteria

Genomic resources for the genus *Bifidobacterium* have expanded considerably in the last decade. More than 215 publicly available genomes representing ∼44 species have now been sequenced, revealing an open pan‐genome of over 30,000 gene clusters and a very small core genome of ∼12 conserved genes, reflecting extensive ecological diversification across the genus (Sharma et al. 2018). Despite this breadth, only a small fraction of sequenced strains originates from milk or fermented dairy products, underscoring a major knowledge gap and reinforcing the importance of TFDPs as underexplored reservoirs of potentially novel or functionally distinct bifidobacteria.

Several studies have reported the presence of *Bifidobacterium* strains in TFDPs, yet the depth of functional, probiotic‐relevant characterization varies considerably. Table 4 summarizes key strains isolated from raw milk, artisanal products, or controlled fermentations, along with their functional traits, ecological origin, and levels of scientific validation.

**TABLE 4:** Probiotic potential of *Bifidobacterium* spp. isolated from milk and traditional fermented dairy products (TFDPs).

Strain	Source/product	Country/region	Fermentation type	Assessed probiotic/functional traits	Validation level	Key findings	Limitations	References
*Bifidobacterium* spp. BT‐14, BT‐9, BT‐39, BT‐27, BT‐2 and BT‐31 (homologous to *B. longum* BB536, *B. bifidum* BGN4, and *B. infantis* ATCC 15697 respectively)	Traditional dairy products	Iran	Spontaneous lactic acid	in vitro assessment of adhesion ability, resistance to gastrointestinal fluids, antimicrobial activity, cholesterol‐ and triglyceride‐lowering ability and antibiotic susceptibility, haemolytic activity in vivo assessment of serum lipid profile, liver tissue lipids, hepatic enzyme profile, faecal cholic acid, total faecal bacteria and gene expression levels	High (in vitro + in vivo)	in vitro activity in lowering triglyceride and cholesterol, tolerance to simulated gastrointestinal juice, the ability to adhere to Caco‐2 cells, acceptable antibiotic susceptibility, and a broad spectrum of antibacterial activity in vivo significant reduction of serum total cholesterol (TC), triglyceride (TG), low‐density lipoprotein cholesterol (LDL‐C), liver tissue lipid levels, and hepatic enzymes	Use of Wistar rats presents limitations, as effects in animals may not fully translate to humans; thus, placebo‐controlled clinical trials are needed to confirm the strains' effectiveness and safety in treating hyperlipidaemia	Afshar et al. (2024)
*Bifidobacterium* spp. *B. breve* (4), *B. longum* (2), *B. bifidum* (2)	Traditional dairy products	Saudi Arabia	Undefined lactic acid	Acid and bile tolerance, adhesion ability	Medium (in vitro)	One strain each of *B. breve* and *B. bifidum* demonstrated strong stress resistance and antimicrobial activity categorised as promising probiotic candidates	No in vivo validation Functional properties not assessed Regional focus Conference abstract format Identified by biochemical kit API 50 CHL only	Al‐Hindi (2013)
*B. animalis* subsp. *lactis* BB‐12	Chr. Hansn's collection of dairy cultures	Denmark	Controlled lactic acid	Acid and bile tolerance, bile salt hydrolase, mucus adhesion, pathogen inhibition, barrier function enhancement, immune interactions	High (clinical + in vitro)	Proven potential probiotic efficacy on the host in clinical studies	Strain‐Specific and Context‐dependent Effects	Jungersen et al. (2014)
*B. crudilactis*	French raw milk cheese	Belgium	Spontaneous lactic acid	Resistance to gastric, pancreatic juices and bile salts	Medium (in vitro)	Ability to grow in raw milk cheese and resist gastrointestinal conditions suggests their possible use as probiotic strains. Potential contribution to flavor development and texture in raw milk cheeses.	Functional properties not assessed Safety not evaluated Limited product scope No in vivo validation	Delcenserie et al. (2013)
*B. mongoliense*			Undefined		Low (presence only)			
*B. longum* B‐2, B‐5, B‐11, B‐19, B‐28	Raw camel milk	Punjab, Pakistan	Spontaneous lactic acid	Survival under gastrointestinal conditions and phenol tolerance, production of EPS, auto‐aggregation ability, cell surface hydrophobicity, DPPH free radical scavenging activity, resistance to hydrogen peroxide, depletion of sodium nitrite, antibacterial activity, cholesterol reduction assay	Medium‐High (in vitro)	*B. longum* B‐11 meets key probiotic criteria: survival in gastrointestinal conditions, safety, functional benefits, and intestinal colonization potential‐making it a strong candidate for use in functional foods and dietary supplements.	Limited to in vivo validation (attachment to rat intestine) No clinical validation Narrow strain selection Regional focus	Yasmin et al. (2020)
*B. aquikefiri*	Water kefir	Belgium	Yeast–lactic acid	Enzyme activities and acid production from different substrates	Low (genomic + phenotypic)	Identified as psychrotrophic bifidobacteria that adapt to low‐temperature fermentations	Functional properties not assessed Safety not evaluated Limited product scope No in vivo validation Regional focus	Laureys et al. (2016)
*Bifidobacterium* spp. FTDC 8943 and *B. longum* FTDC 8643	Local dairy products	Penang, Malaysia	Lactic acid	Adhesion to intestinal epithelial cells, acid tolerance, bile tolerance, antimicrobial activity	Medium (in vitro)	Showed good colonization and adhesion to mucin, tolerance to acid and bile, as well as the production of potential antimicrobial substances toward certain enteric pathogens	No in vivo validation Safety not evaluated	Tham et al. (2012)
*B. lactis* DR10	Fermented milk product	New Zealand	Controlled lactic acid	Adhesion to human intestinal cell line including HT‐29, Caco‐2, and HT29‐MTX antagonistic activity against enterotoxigenic *E. coli*	Medium (in vitro)	It is assumed that organic acids and proteinaceous factors produced by *B. longum* BL2928 worked together in vivo to interfere with the binding of *E. coli *strains to intestinal epithelium	No in vivo validation Functional properties not assessed Safety not evaluated	Gopal et al. (2001)

A critical analysis of these studies reveals that only a minority of isolates have undergone both in vitro and in vivo validation, an essential benchmark for establishing robust probiotic potential. The BT‐series isolates from Iranian traditional dairy products (Collado et al. 2007) represent a rare example of comprehensive characterization demonstrating acid/bile resistance, adhesion ability, antimicrobial activity, cholesterol reduction, and host‐modulatory effects in a rat model. Likewise, *B. animalis* subsp. *lactis* BB‑12 remains one of the best‐validated strains globally, supported by extensive human and preclinical data demonstrating consistent functional and safety profiles (Jungersen et al. 2014).

This inconsistency in scientific validation also highlights a key conceptual limitation. Most studies prioritize detection or preliminary functional assays, while only a small number progress to strain‐level identification, ecological classification, or safety and viability assessments in real food matrices. Such variation reduces cross‐study comparability and constrains the ability to determine which isolates possess genuine translational potential. Furthermore, the lack of standardized protocols for isolation, viability tracking, and genome‐based confirmation limits the establishment of strain provenance across different TFDP ecosystems.

In contrast, several studies document promising traits such as mucin adhesion or pathogen inhibition based exclusively on in vitro assays. Examples include isolates from camel milk (Yasmin et al. 2020), Malaysian dairy products (Tham et al. 2012), and Saudi artisanal samples (Al‐Hindi 2013). Although informative, these studies do not test survival or colonization in vivo, leaving uncertainty about gastrointestinal survival, host interaction, and relevance beyond in vitro screening.

Other taxa, such as *B. mongoliense* or *B. aquikefiri*, have been identified through culture‐independent or genomic surveys but lack phenotypic characterization. Although their detection suggests that bifidobacteria may persist in specific fermentation environments, especially in raw or spontaneously fermented products, the absence of functional data limits the ability to assess their candidacy for probiotic development and hinders translational potential.

The link between strain source and fermentation type is also revealing. Spontaneously fermented products, particularly raw milk or camel milk‐based systems, appear to harbor a broader spectrum of bifidobacteria, possibly due to minimal processing and diverse microbial inputs. However, the frequent absence of strain‐level typing or reproducible isolation protocols limits the ecological interpretation (Fugl et al. 2017; Yu et al. 2021). Conversely, controlled fermentations using commercial starters yield better‐characterized strains but may lack ecological diversity or novelty.

Overall, Table 4 highlights an imbalance in the field: most studies fall within a medium‐level validation, with limited progression to clinical or in vivo confirmation. This reflects both methodological challenges and a historical focus on presence/absence data rather than functional characterization.

To advance bifidobacterial research in TFDPs, future studies must (i) adopt strain‐resolved multi‐omics (metagenomics, transcriptomics, and metabolomics); (ii) validate survival and metabolic activity using in vitro–in vivo pipelines; and (iii) contextualize findings within the ecological conditions of each fermentation type (e.g., pH, oxygen, and microbial competition as summarized in Figure 2). Such a framework would enable progression from isolated reports to a systems‐level understanding of how traditional dairy environments should be considered as reservoirs of functionally relevant *Bifidobacterium* strains with potential for downstream isolation and validation.

## 4 Unlocking Probiotic‐Relevant Potential of Bifidobacteria in Traditional Dairy: Advances in Detection and Characterization

As discussed in Sections 2 and 3, the ecological status, functional relevance, and strain‐level validity of bifidobacteria detected in TFDPs remain uncertain. Reports of presence alone do not establish ecological integration, viability, or probiotic potential. Addressing these limitations requires methodological approaches capable of moving beyond detection toward functional and translational validation.

Accordingly, this section examines how analytical advances, from classical isolation techniques to multi‐omics platforms, contribute to resolving the ecological ambiguities and validation gaps previously identified. Rather than presenting tools in isolation, the discussion follows a progression from detection to strain‐level characterization and finally to application‐oriented selection, aligning methodological capacity with the biological and technological questions outlined earlier in the review.

### 4.1 From Traditional Isolation to Modern Tools: Methodological Transitions

Early studies relied almost exclusively on culture‐dependent techniques, using selective anaerobic media to isolate *Bifidobacterium* from milk and fermented dairy. These methods were effective for detecting dominant or well‐characterized species but often failed to capture the full microbial spectrum, particularly slow‐growing, low‐abundance, or unculturable strains (Ventura et al. 2007). As a result, earlier reports likely underestimated the prevalence and diversity of bifidobacteria in artisanal products.

With the advent of culture‐independent techniques, particularly 16S rRNA amplicon sequencing, the detection landscape changed dramatically. Researchers could now profile entire microbial communities without cultivation, allowing for more consistent detection of *Bifidobacterium* in TFDPs where they had previously gone unnoticed. High‐throughput platforms enabled the identification of multiple species, including *B. breve*, *B. adolescentis*, and *B. longum*, not only in human‐associated niches but also in camel milk, fermented curds, and kefir‐like products (Milani et al. 2017; Turroni et al. 2012).

However, these methods have their limitations. Amplicon‐based studies lack resolution at the strain level and provide little information on metabolic activity or ecological function. This has led to a shift toward metagenomic and genomic tools, capable of providing both taxonomic depth and functional insight. These methodological transitions directly address the detection limitations discussed in Section 2, particularly the difficulty of distinguishing transient DNA signals from viable, ecologically integrated bifidobacterial populations.

### 4.2 Shotgun Metagenomics: Profiling Functional Gene Potentials in Traditional Fermented Dairy Products (TFDPs)

Building upon the need for strain‐level ecological validation highlighted in Section 3, shotgun metagenomics enables not only taxonomic identification but also functional inference within complex TFDP matrices. Shotgun metagenomics overcomes many of the limitations of earlier tools by enabling whole‐community profiling at the species and strain levels while also revealing the genetic potential of resident microbes. In the context of TFDPs, this means moving from detection to inference of functional potential. For example, genes associated with carbohydrate utilization, EPS production, bacteriocin synthesis, and SCFA biosynthesis have been detected in *Bifidobacterium*‐dominated metagenomes from spontaneously fermented products (Almeida et al. 2019; O'Callaghan and van Sinderen 2016). These traits are linked to functions relevant to both probiotic development and technological performance (e.g., texture, stability, and flavor development).

Such findings offer early but compelling evidence that traditional dairy matrices can harbor functionally competent *Bifidobacterium* strains, especially when fermentation conditions (e.g., pH, oxygen tension, substrate availability) align with their ecological preferences, as shown in Figure 2. Still, most TFDP‐focused metagenomic studies remain exploratory. Without paired culture‐based isolation, transcriptomic validation, or in vivo studies, the functional relevance of these detected traits remains provisional.

### 4.3 Whole Genome Sequencing: From Gene Presence to Safety and Strain Designation

While metagenomics clarifies community‐level potential, whole genome sequencing (WGS) is essential for resolving strain‐level identity, safety, and functional credibility, a prerequisite for moving from ecological detection to probiotic candidacy (Li et al. 2025). WGS has become the gold standard for strain‐level characterization. It allows researchers to do the following:

- Confirm genetic determinants associated with probiotic functionality (e.g., acid/bile tolerance, adhesion factors, vitamin biosynthesis),
- Screen for undesirable genes (e.g., antimicrobial resistance determinants, virulence‐associated genes),
- Compare functional pathways across *Bifidobacterium* strains from different origins (dairy, gut, honeybee, environment), and
- Establish phylogenetic links between strains isolated from TFDPs and strains used in probiotic products.

For example, WGS has shown that certain *B. animalis* subsp. *lactis* strains used in fermented dairy differ markedly in EPS operons and adhesion islands, directly affecting their performance in both food matrices and host systems (Bottacini et al. 2014). Similar analyses are emerging for *B. mongoliense* and *B. pseudolongum* strains recovered from traditional Asian fermented milks, though full safety profiles remain incomplete. Importantly, WGS also supports rational selection of candidate strains for product development, linking TFDP‐derived isolates to specific probiotic or techno‐functional outcomes. As Table 4 shows, most isolates have been evaluated primarily through in vitro assays, whereas only a limited number, including well‐characterized strains such as *B. animalis *subsp. *lactis* BB‐12 has undergone comprehensive genomic characterization and functional validation.

### 4.4 Methodological and Ecological Challenges in Traditional Fermented Dairy Products (TFDPs) Microbiome Characterization

Despite notable progress in sequencing technologies and culture‐omics strategies, studying *Bifidobacterium* within TFDPs presents several methodological, ecological, and interpretative challenges. Importantly, culture‐dependent and molecular approaches provide complementary and mutually informative insights: sequencing‐based methods reveal community structure and diversity, while cultivation remains essential for confirming microbial viability and enabling functional characterization of candidate strains. These obstacles are not only technical but also conceptual, stemming from the intrinsic variability of traditional fermentation systems, the physiological nature of bifidobacteria, and limitations in current analytical pipelines.

Sampling inconsistency remains a major barrier. Many TFDPs are produced in artisanal or rural settings without sterile handling, controlled temperature, or standardized equipment. As a result, microbial populations may shift during transport or storage before analysis, making it difficult to distinguish live and active microbes from damaged or dormant cells (Giraffa, 2004). Sample degradation can obscure low‐abundance but biologically relevant taxa such as bifidobacteria, leading to underestimation of their presence and ecological contribution.

The oxygen sensitivity and slow growth rate of *Bifidobacterium* further complicate isolation efforts. Strict anaerobic conditions are required to prevent viability loss during culturing, yet such conditions are rarely feasible during initial fermentation, sampling, or laboratory processing. In mixed TFDP communities dominated by rapidly growing LAB, bifidobacteria are easily outcompeted, often leading to false negatives in culture‐based studies even when molecular evidence indicates their presence.

Fermentation heterogeneity also poses a significant challenge. Batch‐to‐batch variation due to differences in milk origin, handling practices, vessel type, fermentation duration, and back‐slopping traditions leads to inconsistent microbial profiles within the same product type (Tigga et al. 2025; You et al. 2024). These variations make it difficult to determine whether detected bifidobacteria are stable ecosystem members, transient entrants, or contamination introduced during processing or sampling. Without repeated batch analysis or strain‐level tracking, ecological significance cannot be confirmed.

Limitations in molecular methods further constrain strain‐level interpretation. While 16S rRNA amplicon sequencing is widely used, it cannot reliably resolve bifidobacteria beyond the genus level, nor can it determine metabolic functionality, viability, or ecological role. Even shotgun metagenomics may fail to capture strain‐level dynamics in low‐abundance populations unless complemented by targeted enrichment, genome binning, or long‐read sequencing approaches. Moreover, DNA‐based methods cannot differentiate live from dead or metabolically active cells, highlighting the need for complementary tools such as viability qPCR, PMA‐based assays (e.g., propidium monoazide treatment prior to PCR to selectively inhibit amplification of DNA from membrane‐compromised cells), transcriptomics, and metabolomics (Emerson et al. 2017; Fujimoto and Watanabe 2013; García‐Cayuela et al. 2009).

An additional conceptual gap is the limited understanding of niche adaptation and matrix‐specific selection pressures. TFDP‐associated bifidobacteria may exhibit unique ecotypes shaped by the milk environment, interaction with LAB consortia, or exposure to fermentation metabolites; however, current methods seldom capture these microecological dynamics. Mapping spatial distribution, micro‐niche preferences, and metabolic cross‐feeding would provide deeper insight into functional integration rather than mere presence.

Addressing these methodological and ecological challenges will require integrated multi‐omics, spatial microbiology, and comparative ecological modeling approaches. Establishing curated reference genomes from TFDP‐derived bifidobacteria, improving anaerobic culture workflows, and applying functional viability assays will be essential to move beyond descriptive detection and toward meaningful ecological and technological characterization.

### 4.5 From Detection to Application: Integrating Genomics With Probiotic Development

Having addressed detection and characterization, the remaining challenge lies in translating genomic potential into validated probiotic function within real TFDP systems. As summarized in Table 4 and expanded in Section 4.6, several *Bifidobacterium* strains isolated from TFDPs, particularly those from camel milk, kefir, or artisanal curd, have demonstrated traits associated with probiotic functionality in screening assays. However, only a subset of these have progressed to strain‐level validation through WGS or in vivo studies, or clinical evaluation. This gap reflects multiple constrains, including prioritization of isolates with strong initial phenotypes, the technical and infrastructural demands of genomic and animal studies, regulatory requirements governing probiotic development, and the complexity of demonstrating functional efficacy within heterogenous food matrices. Collectively, these challenges highlight that the transition from microbial detection to validated probiotic application remains a critical bottleneck.

Linking genomic traits (e.g., SCFA pathways, adhesion genes, EPS clusters) with phenotypic outcomes (e.g., pathogen inhibition, colonization, texture enhancement) provides a rational basis for selecting candidate strains. This integrative approach enables the identification of bifidobacterial strains that are not only viable but also functionally compatible with specific food matrices and technological processes.

Approaches such as in situ enrichment of native bifidobacterial populations within TFDP matrices may enhance their relative abundance or metabolic activity; however, such strategies should be interpreted within the context of functional food development rather than probiotic designation. From a translational and regulatory perspective, the isolation, strain‐level characterization, and reintroduction of selected bifidobacteria into controlled food systems remain the primary pathway for developing validated probiotic products (Hill et al. 2014; Sanders et al. 2018).

Following isolation and validation, candidate bifidobacterial strains may be incorporated into controlled fermentation systems designed to support their viability and functional performance. In this context, optimization strategies should focus on previously isolated and validated strains rather than native microbial communities. These may include the following:

- enriching the growth and metabolic activity of validated bifidobacterial strains through targeted prebiotic supplementation (e.g., galacto‐oligosaccharides, fructo‐oligosaccharides, or milk‐derived oligosaccharides),
- co‐fermentation with synergistic microorganisms (e.g., LAB or yeasts) to improve stability, viability, and functional performance of introduced strains, an approach explored in commercial and pilot‐scale dairy production in countries including Germany, China, the United States, and Australia (Anumudu et al. 2024; Geng et al. 2026; Sun et al. 2025).
- Engineering fermentation parameters (e.g., reducing oxygen, lowering redox potential, and controlled pH modulation) to support survival, metabolic activity, and functional expression of selected strains.

Importantly, such strategies are applicable to controlled or reformulated systems inspired by TFDPs, rather than traditional products themselves, which remain inherently variable and are not suitable for direct probiotic designation under current regulatory frameworks.

These translational frameworks enable progression beyond descriptive detection toward design and evaluation of scientifically validated food systems incorporating defined probiotic strains. The intersection of TFDP microbial diversity with advanced sequencing and characterization tools presents a significant opportunity to identify novel bifidobacterial candidates. However, functional validation, including strain‐level characterization, viability assessment, and clinical substantiation, remains the critical step in realizing this potential. By integrating detection, characterization, and ecological insight within a structured translational pathway, TFDPs can be effectively leveraged as reservoirs for the discovery of bifidobacterial strains with relevance for next‐generation functional foods and probiotic applications.

### 4.6 Advances in Isolation Strategies and Metabolomics for Validating TFDP‐Derived Bifidobacteria

Although culture‐independent methods have transformed detection, the isolation of bifidobacteria from TFDPs remains a critical bottleneck. Their low abundance, oxygen sensitivity, and competition from rapidly growing LAB often hinder successful recovery (Roy 2001; Tham et al. 2012). Recent advances, including culturomics‐based anaerobic workflows, selective enrichment strategies; modified media such as BIM‐25 or mMRS; colony‐PCR screening; and MALDI‐TOF identification, have begun to improve isolation success rates from complex dairy matrices (Howe et al. 2024; Nebra and Blanch 1999; Wang et al. 2024). These approaches are particularly relevant for bifidobacteria. Modified media such as BIM‐25 or mMRS are designed to favor the growth of bifidobacteria while suppressing competing LAB and other background microbiota, thereby improving recovery of low‐abundance populations (Nebra and Blanch 1999; Roy 2001). Anaerobic culturing workflows further enhance isolation by accommodating the oxygen‐sensitive physiology of bifidobacteria (Tham et al. 2012). In addition, colony‐PCR screening and MALDI‐TOF identification enable rapid discrimination of bifidobacterial isolates from morphologically similar LAB, improving isolation efficiency and accuracy (Howe et al. 2024; Wang et al. 2024).

Importantly, in silico predictions derived from metagenomics or whole‐genome analyses must be confirmed through wet‐lab validation, as gene presence does not necessarily indicate phenotypic expression (Ayyash et al. 2021; Bottacini et al. 2014; Ruiz et al. 2013). Key traits, including acid and bile tolerance (Ruiz et al. 2013), adhesion (Westermann et al. 2016), EPS production (Sadeghi et al. 2024), antimicrobial activity, and metabolic outputs, still require verification through targeted assays and, ideally, clinical study involving animal or human trials.

Metabolomics has also emerged as a powerful complementary tool, providing direct evidence of metabolic functionality. Untargeted high‐resolution LC‐MS platforms, such as Orbitrap or quadrupole time‐of‐flight (QTOF) systems, enable comprehensive profiling of metabolites produced during fermentation, while targeted LC‐MS/MS or GC‐MS approaches (e.g., multiple reaction monitoring or selected ion monitoring) allow accurate quantification of specific compounds. These methods have been used to characterize metabolites including SCFAs, organic acids, amino‐acid derivatives, flavor‐active volatiles, and other postbiotic compounds produced during traditional dairy fermentation (Caboni et al. 2019; Rehman et al. 2024; Sun et al. 2021).

In the context of bifidobacterial research, such metabolomic approaches provide functional evidence of metabolic activity and help link strain‐level presence to ecological performance and technological relevance. Importantly, these metabolites also influence sensory attributes such as flavor, aroma, and texture, making metabolomics particularly valuable for connecting microbial function with product quality in TFDP systems.

Collectively, the integration of anaerobic culturing, strain‐resolved genomics, viability assays, and metabolomics provides a pathway for transforming descriptive reports into evidence‐based identification of functionally relevant bifidobacteria. When aligned with the ecological framework established in Section 2 and the functional criteria outlined in Section 3, these tools allow TFDPs to be evaluated not merely as fermented foods but as dynamic microbial ecosystems with potential to yield rigorously validated bifidobacterial strains for future functional applications.

## 5 Challenges and Limitations

Despite growing interest in TFDPs as potential sources of bifidobacteria, the translation of their presence into validated probiotic applications remains constrained by a set of interrelated ecological, methodological, technological, and regulatory challenges. These constraints do not operate independently; rather, they form a coupled system in which limitations in detection, viability, functional validation, and regulatory compliance reinforce one another (Hill et al. 2014; Sanders et al. 2018; Sibanda et al. 2024). As a result, the pathway from ecological occurrence to probiotic application remains fragmented, highlighting a fundamental disconnect between microbial discovery in complex fermentation systems and the stringent requirements for probiotic designation (O'Callaghan and van Sinderen 2016; Turroni et al. 2014).

A central limitation lies in the distinction between microbial detection and functional relevance. Many studies report the presence of bifidobacteria in TFDPs using culture‐dependent or molecular approaches, yet detection alone does not confirm viability, metabolic activity, or ecological integration (Lee and O'Sullivan 2010; Rivière et al. 2016). DNA‐based methods may detect low‐abundance or non‐viable populations, while culture‐based approaches often underestimate diversity due to the fastidious and anaerobic nature of bifidobacteria (O'Callaghan and van Sinderen 2016). This methodological gap complicates interpretation and limits the ability to distinguish between active community members and transient or incidental populations.

Closely linked to detection is the challenge of viability and functional persistence across the product lifecycle. Even when viable cells are detected at the end of fermentation, their survival during storage and at the point of consumption is rarely assessed. Moreover, probiotic functionality requires not only survival but also retention of metabolic activity and sufficient viable counts at consumption, criteria that are seldom verified in TFDP studies (Sibanda et al. 2024; Schöpping et al. 2022).

Strain‐level resolution represents another critical bottleneck. Traits associated with probiotic functionality are inherently strain‐specific, yet many studies report bifidobacteria at the genus or species level without genomic confirmation or phenotypic validation (Hill et al. 2014; Sanders et al. 2018). This lack of resolution limits the ability to distinguish between functionally relevant strains and those that are ecologically incidental.

These challenges are further compounded by ecological ambiguity. TFDPs are open and heterogeneous systems influenced by raw materials, environmental microbiota, artisanal practices, and batch variability. This complexity makes it difficult to determine whether detected bifidobacteria are stable community members or transient populations (Rivière et al. 2016).

Technological and industrial constraints further limit translation. Even when promising strains are identified, their incorporation into dairy systems is challenged by oxygen sensitivity, stress intolerance, and incompatibility with standard processing conditions (Sibanda et al. 2024). At the same time, regulatory frameworks require strain‐level identification, safety validation, and clinical evidence of health benefit, criteria that TFDP‐derived strains rarely meet (Hill et al. 2014; Sanders et al. 2018).

Taken together, these interconnected challenges create a bottleneck that restricts the progression from exploratory identification of bifidobacteria in TFDPs to their development as validated probiotic strains.

### 5.1 Viability and Functional Integrity across Traditional Fermented Dairy Products (TFDPs) Lifecycle

As outlined above in Sections 3 and 4, viability represents a central constraint linking ecological detection to functional relevance. A persistent challenge in deploying bifidobacteria in TFDPs is ensuring viability throughout production, storage, and consumption. Many strains identified in artisanal dairy matrices are sensitive to environmental stresses common in traditional settings, such as oxygen exposure, thermal instability, acidic pH, and absence of cold chain infrastructure (Ku et al. 2024; Schöpping et al. 2022). Unlike LAB species, bifidobacteria generally lack robust stress‐response systems, rendering them susceptible to functional loss even when they remain viable. This has important implications for their potential probiotic efficacy, as a threshold of 10^6^ CFU/g at the point of consumption is widely accepted as a minimum concentration often cited for potential probiotic efficacy in regulated products (Habiba et al. 2025; Uhegwu and Anumudu 2025).

Few TFDP studies evaluate microbial viability longitudinally or verify whether detected bifidobacteria remain viable and functionally active at the point of consumption; most assess survival only at the end of fermentation, not after packaging, during storage or during gastrointestinal transit, making functional persistence uncertain (Table 4). As outlined in Table 4, the majority of bifidobacterial isolates reported from TFDPs have been evaluated only through in vitro assays, with relatively few progressing to in vivo validation and only one strain (*B. animalis* subsp. *lactis* BB‐12) supported by clinical evidence. Several isolates demonstrate acid and bile tolerance, adhesion capacity, antimicrobial activity, or EPS production under laboratory conditions; however, these assessments rarely extend to strain‐level genomic confirmation or longitudinal viability tracking within the original product matrix. Notably, most published studies do not report the proportion of samples yielding bifidobacteria, making it difficult to derive comparable isolation‐rate statistics across TFDPs.

Studies on related dairy systems, including yogurt and kefir, similarly report strain‐dependent survival during storage, with viability often declining over time (Desfossés‐Foucault et al. 2012; Matias et al. 2016; Meng et al. 2010). Although novel strains have been isolated from traditional fermentations such as water kefir (Breselge et al. 2024; Laureys et al. 2016), longitudinal persistence and functional validation remain limited.

Collectively, these findings indicate that while TFDP‐derived bifidobacteria may exhibit promising functional traits, robust evidence of sustained viability and validated probiotic performance across the product lifecycle remains scarce. Protective approaches, such as microencapsulation, oxygen‐scavenging LAB or prebiotic incorporation, have shown potential to enhance survival in fermented dairy matrices (Meybodi et al. 2020; Norouzbeigi et al. 2021). However, these approaches are rarely evaluated within authentic TFDP contexts, where artisanal variability and process heterogeneity further complicate reproducibility. Addressing these gaps will require systematic longitudinal studies integrating viable counts, functional assays, and genomic validation across production and storage phases to substantiate the probiotic integrity of TFDP‐derived bifidobacteria.

### 5.2 Strain‐Specific Variability and Ecological Ambiguity

In addition to viability, strain‐level variability further complicates the interpretation of bifidobacterial presence. Traits associated with probiotic functionality in *Bifidobacterium* are highly strain‐specific, with significant intra‐species variability in functional properties like acid/bile resistance, SCFA production, epithelial adhesion, and immunomodulation capacity (Hill et al. 2014; O'Callaghan and van Sinderen 2016; Sanders et al. 2018). This creates a paradox in TFDP research: although several strains isolated from fermented camel milk, kefir, or dahi exhibit promising in vitro phenotypes (see Table 4), most lack comprehensive genomic, phenotypic, and safety validation for probiotic designation. As a result, strains may appear functionally promising at the laboratory level while remaining translationally uncertain. Consequently, probiotic designation of TFDP‐derived strains remains provisional until supported by strain‐resolved genomic characterization and human clinical evidence.

In addition, the ecological integration of TFDP‐associated bifidobacteria remains incompletely resolved. As noted earlier, distinguishing stable fermentation residents from transient or non‐viable populations requires longitudinal, strain‐level tracking within authentic production systems. Resolving this uncertainty is essential to substantiate functional relevance within traditional dairy matrices.

### 5.3 Regulatory Hurdles and Labeling Constraints

These scientific limitations are further reinforced by regulatory requirements governing probiotic designation. The global regulatory landscape governing probiotic claims remains fragmented, with country‐specific requirements for strain documentation, functionality, and safety that rarely accommodate the variability and lack of standardization inherent to traditional fermented products (Bhardwaj et al. 2025; Mukherjee et al. 2022). Regulatory oversight in regions such as the United States, the European Union, and Australia is administered by the US food and drug administration (FDA), the European Food Safety Authority (EFSA), and Food Standards Australia New Zealand (FSANZ), respectively. Comparable regulatory authorities operate in other jurisdictions, including Health Canada and the National Medical Products Administration (China), although evidentiary requirements and approval pathways vary across regions.

These authorities require strain‐level identification, validated functional claims, and demonstrated safety through in vivo studies and genome sequencing before probiotic designation or health claims can be approved (Komala et al. 2023; Tan et al. 2024). Very few TFDP‐derived strains reported in the literature meet these standards, and none of the studies in Table 4 provide regulatory‐ready dossiers. Efforts to harmonise probiotic guidelines, including initiatives under the Codex Alimentarius Commission, remain non‐binding and largely focused on industrially produced probiotic foods (Bhardwaj et al. 2025; Roe et al. 2022). This regulatory gap limits the ability to label TFDPs as probiotic products or to support health claims in international markets. Most TFDPs are produced in non‐industrial settings with limited documentation, traceability, and process control, creating a mismatch between traditional practices and modern regulatory expectations. Labeling presents an additional challenge, as small‐scale producers rarely have the capacity to guarantee the presence and stability of specific strains at efficacious levels throughout a product's shelf life (Bentahar et al. 2024; Colautti et al. 2024). Calls for greater rigor in quality control and labeling accuracy have been made to ensure probiotic safety, viability, and efficacy (International Probiotics Association, 2017; Kolaček et al. 2017).

### 5.4 Research Gaps and Translational Bottlenecks

Collectively, these challenges highlight several key research gaps that must be addressed. Despite increasing interest, TFDP‐focused research remains geographically narrow and methodologically uneven. Studies are disproportionately concentrated in South and Central Asia, with underrepresentation from traditional dairy systems in the Middle East, Africa, Eastern Europe, or Latin America. Even where *Bifidobacterium* detection has occurred, functional and clinically relevant data are sparse, with most studies relying on PCR‐based presence/absence or culture‐based enumeration without deeper validation. This limitation reflects several factors, including the technical challenges associated with isolating low‐abundance obligate anaerobes from complex dairy matrices, limited access to genomic and animal‐model facilities in regions where TFDPs are traditionally produced, and the high financial and regulatory burden associated with conducting human clinical trials. Consequently, research efforts have often prioritized detection and preliminary phenotypic screening over comprehensive translational validation.

Table 4 reveals that less than a third of studies employed genomic tools, and even fewer performed phenotypic assays such as adhesion, antimicrobial activity, or SCFA profiling. Human clinical validation, a key requirement for probiotic designation, is virtually absent. Additionally, batch reproducibility, matrix‐strain interactions, and long‐term stability have received minimal attention, despite being critical for industrial translation.

Technologically, the scale‐up of TFDPs poses inherent challenges: maintaining microbial diversity, ensuring quality control, and preserving bifidobacterial viability are difficult in large‐volume or export‐oriented formats. These bottlenecks require engineering solutions (e.g., oxygen control, modular fermentation), but such innovations are rarely tested in TFDP contexts. Additionally, translating TFDP‐derived bifidobacteria into validated probiotic products or industrial applications requires rethinking conventional scale‐up strategies, since characteristics such as strain sensitivity, matrix dependency, and ecological variability may not align with highly standardized commercial production workflows. Developing flexible, modular fermentation strategies that preserve microbial diversity while ensuring safety and viability will therefore be key to bridging the gap between artisanal origin and industrial applicability.

### 5.5 Toward Sustainable and Culturally Rooted Probiotic Solutions

Amidst these limitations, the emerging genomic and functional insights into TFDP‐derived *Bifidobacterium* strains offer cause for optimism. As outlined by O'Callaghan and van Sinderen (2016) and Turroni et al. (2014), a systems‐level approach linking metagenomics, strain‐level genomics, and fermentation ecology can help identify strains best suited for functional, stable, and scalable applications.

In summary, TFDPs represent promising but under‐validated reservoirs of bifidobacteria, with substantial limitations spanning viability, strain resolution, regulatory compliance, and translational scalability. While numerous studies report the presence of bifidobacteria and associated functional traits, these observations remain largely disconnected from robust validation frameworks required for probiotic development.

A key unifying challenge is the fragmentation of evidence, where ecological detection, functional characterization, and clinical validation are rarely integrated within a single framework. This limits the ability to establish causal links between microbial presence, functional performance, and health outcomes.

Beyond these constraints, the presence of bifidobacteria in TFDPs should be interpreted as an ecological signal rather than evidence of probiotic functionality per se. The critical question is therefore not simply whether bifidobacteria are present, but whether they represent stable, functionally active members of fermentation ecosystems capable of meeting probiotic criteria.

Addressing this challenge will require coordinated approaches integrating ecological analysis, strain‐resolved genomics, functional validation, and controlled application studies. Such integration is essential to move TFDP‐derived bifidobacteria from descriptive occurrence toward evidence‐based probiotic development.

## 6 Future Perspectives

A key challenge moving forward is to transition from descriptive detection of bifidobacteria in TFDPs to evidence‐based identification of functionally relevant strains. This will require the development of integrated validation pipelines that systematically connect ecological data with functional and clinical outcomes. Such pipelines should combine strain‐resolved metagenomics, viability assessment, metabolomic profiling, and controlled in vitro and in vivo validation. Importantly, this approach shifts the focus from “presence‐driven” discovery to “function‐driven” selection, enabling a more targeted and translationally relevant exploration of TFDP‐derived bifidobacteria.

This review positions TFDPs not as probiotic foods per se, but as valuable ecological reservoirs for the identification of bifidobacterial strains with translational potential. Future research should prioritize the isolation, strain‐level validation, and controlled application of these microorganisms within defined food systems, alongside optimization of carrier matrices to ensure viability, functionality, and regulatory compliance. Strategies based solely on in situ enrichment of native bifidobacterial populations should be interpreted within the context of functional food development rather than probiotic designation, which requires validated strains and controlled delivery systems.

In parallel, research must address strain–matrix compatibility. Rather than pursuing generic probiotic incorporation strategies, future work should focus on fermentation‐type–specific optimization, including co‐culture design, oxygen management, and metabolite modulation, to support the viability and functional performance of validated bifidobacterial strains within defined food matrices.

Advances in culture‐omics, high‐resolution metabolomics, and comparative genomics provide powerful tools to refine strain selection based on ecological fitness and functional performance. Integrating these approaches with longitudinal viability tracking will enable more rigorous assessment of probiotic candidacy under realistic processing and storage conditions.

From an application standpoint, controlled or reformulated systems inspired by TFDPs may serve as platforms for regionally adapted formulations incorporating validated bifidobacterial strains. Such approaches align with precision fermentation strategies and culturally contextualized nutrition innovation, offering pathways for locally developed functional foods that meet regulatory standards without eroding traditional practices.

To operationalize this transition, future work should focus on a set of coordinated priorities, including (i) rigorous clinical validation through strain‐specific, randomized trials aligned with regulatory expectations; (ii) technological optimization strategies to stabilize oxygen‐sensitive bifidobacteria, such as microencapsulation and controlled fermentation conditions; and (iii) longitudinal assessment of strain viability across production, storage, and gastrointestinal simulation. In addition, integrated omics frameworks linking genomic potential to confirmed metabolic and functional outcomes will be essential, alongside cross‐disciplinary collaboration bridging microbiology, food engineering, regulatory science, and socio‐cultural research.

Collectively, these directions outline a shift from descriptive surveys toward evidence‐based development. TFDP‐associated bifidobacteria represent a promising interface between microbial ecology and functional food science; however, their successful translation depends on rigorous strain‐level validation, technological optimization, and alignment with regulatory frameworks governing probiotic application.

## Author Contributions

**Mst. Umme Habiba**: conceptualization, writing – original draft, writing – review and editing, investigation, visualization, methodology, formal analysis, data curation, validation. **Md. Morshedur Rahman**: conceptualization, writing – review and editing. **Mary Ann Augustin**: writing – review and editing. **Cristian Varela**: visualization, writing – review and editing. **Helen Morris**: writing – review and editing. **Hayriye Bozkurt**: conceptualization, writing – original draft, investigation, writing – review and editing, project administration, supervision, funding acquisition, resources, methodology, validation, formal analysis, data curation.

## Conflicts of Interest

The authors declare no conflicts of interest

## Data Availability

All data are presented in the current study.
